# Description of the surgical technique for condylectomy with minimally invasive surgery to treat interdigital helomas on the lesser toes: a Delphi study

**DOI:** 10.1186/s13047-019-0322-5

**Published:** 2019-02-14

**Authors:** Luis M. Marti-Martinez, Alba Gracia-Sánchez, Javier Ferrer-Torregrosa, Rubén Lorca-Gutierrez, Jonatan Garcia-Campos, Salvador Pedro Sánchez-Pérez

**Affiliations:** 1 Behavioural and Health Sciences Department, Miguel Hernandez University, Ctra. Nnal. 332 s/n, 03550 San Juan de Alicante, Spain; 2 University Clinic of Podiatry, Complutense de Madrid University, Av. Seneca, 2, 28040 Madrid, Spain; 3 Physiotherapy and Podiatry Department, Catholic university of Valencia San Vicente Mártir, C/ Ramiro de Maeztu, 14, 46900 Torrente, Spain

**Keywords:** Podiatry, Foot, Surgical procedures, Delphi technique

## Abstract

**Background:**

Descriptions of the techniques for condylectomies via minimally invasive surgery (MIS) to treat interdigital helomas of the lesser toes are scarce in the literature. This study aimed to define and describe this surgical technique.

**Methods:**

This observational study was performed using the Delphi method. We collected the anonymous opinions of a multidisciplinary international panel of ten experts by answering a 43-items questionnaire via e-mail. Statements with an average score ≥ 3 were included in the next round, as were those in which none of the three statements reached the minimum score of 3 within the same item.

**Results:**

Response rate: 90%. Three rounds were needed to reach consensus on proposed items. A new statement that combined two statements was proposed in round 3. Eleven recommendations regarding the incision and instruments used to perform this surgical technique were obtained based on the expert consensus.

**Conclusions:**

A longitudinal incision to the distal pulp of the toe or an incision to the centre of the plantar aspect of the head of the proximal phalanx should be performed according to the affectation, and a Beaver 64 scalpel blade, a blunt elevator and a Shannon-Isham burr are the most acceptable tools for this kind of surgery.

## Background

Foot problems are associated with pain and can affect quality of life [1, 2]. According to a 2015 survey of podiatrists in the United Kingdom [3], hyperkeratosis (calluses) and helomas (corns) were the most frequently treated problems. Helomas are circumscribed hyperkeratotic lesions that are produced by the hyperplasia of the epidermis [4] due to excessive pressure or skin friction in the area. They have a conical central nucleus that deepens towards the dermis, causing pain and frequent inflammation [5]. If the pressure persists at high levels, it will produce inflammation that can form an ulcer. Infection appears in the most severe cases, where the ulceration is subjacent [6].

Helomas are most often found on the lesser toes of the foot. They are categorised as hard corns, also known as heloma durum, or soft corns, also known as heloma molle. A heloma molle is a painful lesion that occurs only interdigitally and is often called an “interdigital heloma”. It is a heloma that has absorbed a considerable amount of moisture from sweat, which leads to a characteristic maceration and sometimes a secondary infection due to fungi or bacteria [5].

Interdigital helomas of the lesser toes tend to occur more frequently in elderly patients [7, 8], with a higher prevalence among women than men [6, 9]. Du [10] and Gillett [11] found that 15% of pathological foot cases are due to interdigital helomas on the lesser toes, that the most frequent place of appearance of interdigital helomas is the fourth interdigital space, and that the interdigital heloma with the highest prevalence is located on the fifth toe at the medial condyle of the distal phalanx. Recent studies have not determined the prevalence of this condition.

Heloma treatment seeks to reduce morbidity and avoid complications [12]. The initial management of lesser toe interdigital helomas is conservative, and surgical treatment is applied only when these treatments fail and the heloma and pain recur [5].

Surgery aims to eliminate the mechanical pressure of the bone structure or the structures involved in the formation of the heloma [5]. Different surgical alternatives exist to treat interdigital heloma including condylectomy, arthroplasty, or syndactyly. The current worldwide surgical treatment trend is to develop and investigate minimally invasive procedures [13]. Minimally invasive surgery (MIS) to the foot uses small-sized incisions between 1 and 4 mm. The bone area to be treated is accessed via these incisions so that the soft tissues receive less dissection and less trauma, thereby causing less postoperative pain and leading to an earlier recovery compared with conventional surgery. In his manual, Cazeau [14] indicated the possibility of treating interdigital helomas with MIS but did not describe the procedure. Descriptions of the techniques for performing condylectomies via MIS on the phalanges of the lesser toes to treat interdigital helomas are scarce in the literature. Only five authors have described this technique: White [15], Hymes [16], Gorman et al. [17], Bycura [18], and, most recently, De Prado et al. [19]. Their techniques are based on their personal preferences, and differences and similarities exist among them.

Consensus methods are being used to solve problems in medicine in order to define levels of agreement on controversial subjects. Through consensus strategies, experts can give the best available information [20]. When the evidence for a treatment is weak or the best way to manage a problem in health remains unclear, a Delphi study could be used in order to develop a treatment protocol [21], or to establish definitions and characteristics of a technique [22, 23]. If there are no clinical guidelines for a non-pharmacological treatment, consensus methods have been used to develop consensus-based practice recommendations for the prescription [24]. Even when some controversy exists regarding the use of an antibiotic, a Delphi study was performed [25]. Although there is no evidence of the reliability of the Delphi method, decisions are strengthened by reasoned argument, the use of participants who have knowledge and an interest in the topic and the use of several rounds helps to increase the validity of the Delphi. However, the validity of results will be ultimately affected by the response rates [26].

Due to the need to review and update the condylectomy via MIS on the phalanges of the lesser toes to treat interdigital helomas, and also the lack of research in this area, we decided to use the Delphi method to define and describe this surgical technique that has agreed expert consensus. Like previous authors, such as Dando et al. [27], we used a structured qualitative technique of professional consensus developed by Dalkey et al. [28].

Given the advances in the anatomical knowledge of the foot and the development of new surgical instruments for the practice of MIS on the foot, it is necessary to review and update the associated surgical techniques. Therefore, this study sought to define and describe the surgical techniques for condylectomy via MIS on the interdigital helomas of the lesser toes.

## Methods

This observational study was conducted between December 12, 2015 and February 12, 2016 using the Delphi method, a systematic and interactive process aimed at obtaining opinions and (if possible) consensus from a group of experts on a topic to improve the effectiveness of decision making in healthcare [26, 29]. The protocol of this study was approved by the Ethics Committee of the institution involved (reference number DPS.LMM.01.16).

### Participants

Initially, a coordinating group was established to select a panel of experts to define the items or aspects needed to describe the technique under study. Their work would be included in a questionnaire used to interpret partial results and issue reports about the results after each round. Furthermore, a final report would be prepared describing the technique for condylectomy via MIS on the interdigital helomas of the lesser toes.

A multidisciplinary international panel of ten experts with accredited experience in MIS of the foot was formed. The panel comprised eight Spanish podiatrist professors of MIS of the foot at Spanish universities. One member was president of the Spanish Association of Minimally Invasive Surgery of the Foot (*Asociación Española de Cirugía de Mínima Incisión del Pie; AEMIS*), two were ex-presidents of the AEMIS, and six were professors accredited by the AAFAS (Academy of Ambulatory Foot & Ankle Surgery). Two panellists were traumatologists (one from Mexico, one from Argentina), one of whom had the title “Distinguished Professor of AAFAS” and was a former vice-president of the AAFAS; the other was a university professor of foot surgery. A lack of agreement on the sample size of Delphi studies exists; however, a minimum of seven and a maximum of 30 is recommended [30].

### Questionnaire

First, the coordinating group defined the items needed to describe the technique in each question (Table 1). To address these items, a structured questionnaire composed of 43 statements was designed based on the surgical techniques described by the authors in their manuals or publications [15–19]. Using this questionnaire, participants reported to what extent they agreed or disagreed with each statement using a five-point Likert scale with the following levels: Strongly disagree/Disagree/Unsure/Agree/Strongly agree). In addition, the questionnaire offered the panellists the possibility of including free text in case they disagreed with any of the statements about a certain item. To make the questionnaire more dynamic, images were added to each statement to clarify the text. The questionnaire was distributed to the experts in January 2016 in an electronic format, which guaranteed the anonymity of the responses.

**Table 1:** Necessary items to describe the surgical technique for condylectomy via MIS on the interdigital helomas of the lesser toes, and the included questions to obtain a consensus

Item objective	Question Number
To identify the most acceptable incision for the resection of the exostosis or the hypertrophic medial or lateral condyle of the distal phalanx.	1, 2, 3 and 4
To identify the most acceptable incision for the resection of the exostosis or the hypertrophic medial or lateral condyle that simultaneously affects the distal phalanx and the middle phalanx on the same side.	5, 6, 7 and 8
To identify the most acceptable incision for the resection of the exostosis or the hypertrophic medial or lateral condyle that simultaneously affects the distal phalanx, the middle phalanx and the head of the proximal phalanx on the same side.	9, 10, 11 and 12
To identify the most acceptable incision for the resection of the exostosis or the hypertrophic medial or lateral condyle that affects the head of the proximal phalanx of the lesser toes.	13, 14, 15 and 16
To identify the most acceptable incision for the resection of the exostosis or the hypertrophic medial or lateral condyle that simultaneously affects the head of the proximal phalanx and the middle phalanx on the same side of the lesser toes.	17, 18, 19 and 20
To identify the most acceptable instrument for performing a minimally invasive skin incision on the interdigital heloma of the lesser toes.	21, 22, 23 and 24
To identify the most acceptable tool for extending the incision until contact with the bone.	25, 26, 27 and 28
To identify the most acceptable instrument for separating adhesions and periosteal elevation.	29, 30, 31 and 32
To identify the most acceptable type of burr for the bone area to perform an osteotripsy.	33, 34 and 35
To identify the most aceptable way to extract the bone paste.	36, 37, 38 and 39
To identify the most aceptable way to perform the closure of the incision.	40, 41, 42 and 43

### Procedure

The experts sent a letter of introduction via e-mail that included the information suggested by Landeta [31] inviting them to participate in the study. After providing consent to participate, the experts completed the electronic questionnaire within 2 weeks after receiving the e-mail. This e-mail provided a brief introduction and the link to the questionnaire. The questionnaires were completed anonymously.

Delphi questionnaire responses were instantly recorded in a Microsoft Excel spreadsheet. After each round, the coordinating group analysed the quantitative and qualitative data (free text). The next round was based on the analysis of the responses received in the previous round. The number of rounds was determined by time taken to reach consensus on each item.

### Data analyses

After each round, the central tendency measure of the values assigned to each item was calculated for every answer. Thus, the median, mean, and standard deviation of each statement were calculated and reordered based on the average values obtained within each item. The coordinating group considered statements with an average of less than 3 within each item as the experts agreeing that this option was not the most appropriate. The three statements were repeated in the next round of the questionnaire for cases in which none of the three statements reached a score of 3. Therefore, in the second round of the questionnaire, statements with an average score equal to or greater than 3 were included, as were those in which none of the three statements reached the minimum score of 3 within the same item. Statements without a clear statistical difference were also included. At the end of each round, a summary of results was sent to the panel of experts. The data were analysed with the SPSS statistical program (SPSS®, Version 22, Chicago (IL), USA).

## Results

Nine experts answered the questionnaire, achieving a response rate of 90%. It took three rounds to reach consensus on all proposed items.

After the first round, 14 statements were excluded, and no comments were received. Table 2 shows the results of this first round. In the second round, the questionnaire was resent to the panellists, excluding the statements that did not reach consensus. After the analysis of the second-round results (Table 3), two statements that did not reach consensus were excluded, as were statements that reached the minimum consensus (average value 3) and repeated the same or increased degree of consensus since the first round. For the third round, the questionnaire included statements 40, 41, 42, and 43 in the same item, included a new statement that combined statements 9 and 11, and proposed two consecutive incisions. The results of the third round are shown in Table 4.

**Table 2:** Delphi round 1 results

Items	Statement	Mean	Median	SD	Second round
1	A longitudinal incision to the distal pulp of the toe	4.00	5.00	1.73	Yes
	A longitudinal dorsal incision to the center of distal phalanx	1.00	1.00	0.00	No
	A plantar incision in the distal phalanx	1.89	1.00	1.36	No
2	A longitudinal incision	4.11	5.00	1.36	Yes
	A longitudinal dorsal incision to the center of middle phalanx	1.00	1.00	0.00	No
	A plantar incision in the middle phalanx	2.22	1.00	1.56	No
3	A longitudinal incision to the distal pulp of the toe	2.88	3.00	1.54	Yes
	A longitudinal dorsal incision to the center of proximal phalanx	1.88	1.00	1.36	No
	A plantar incision in the proximal phalanx	2.78	2.00	1.92	Yes
4	A longitudinal incision	2.33	2.00	1.58	No
	A longitudinal dorsal incision to the center of proximal phalanx	1.56	1.00	1.13	No
	A plantar incision in the proximal phalanx	3.44	4.00	1.59	Yes
5	A longitudinal incision to the distal pulp of the toe	2.44	2.00	1.59	Yes
	A longitudinal dorsal incision to the center of proximal phalanx	1.67	1.00	1.41	No
	A plantar incision in the proximal phalanx	3.78	5.00	1.72	Yes
6	Beaver 64 scalpel blade	3.78	4.00	1.48	Yes
	Beaver 64 MIS scalpel blade	3.44	4.00	1.74	Yes
	Number 15 scalpel blade	1.68	1.00	1.32	No
7	The same scalpel	4.11	5.00	1.17	Yes
	A blunt elevator	3.22	3.00	1.86	Yes
	A rasp	1.22	1.00	0.44	No
8	The same scalpel	3.11	3.00	1.69	Yes
	A blunt elevator	3.44	4.00	1.74	Yes
	A rasp	2.44	2.00	1.42	No
9	A Shannon-Isham burr of an appropriate size	4.56	5.00	1.01	Yes
	A 44 Shannon burr	2.22	1.00	1.72	No
	A rasp	2.00	2.00	3.32	No
10	Manual pressure	4.44	5.00	1.01	Yes
	A saline solution	3.33	3.00	1.66	No
	A rasp	4.44	5.00	0.73	Yes
11	Suture thread	2.89	3.00	1.54	Yes
	Closure strips	1.89	1.00	1.36	Yes
	Bandage pressure	2.78	2.00	1.92	Yes

**Table 3:** Delphi round 2 results

Items	Statement	Mean	Median	SD	Third round
1	A longitudinal incision to the distal pulp of the toe	4.77	5.00	0.66	No
2	A longitudinal incision	4.44	5.00	1.13	No
3	A longitudinal incision to the distal pulp of the toe	2.55	3.00	1.59	Yes
	A plantar incision in the proximal phalanx	3.00	2.00	1.94	Yes
4	A plantar incision in the proximal phalanx	3.33	4.00	1.80	No
5	A longitudinal incision to the distal pulp of the toe	2.11	2.00	1.45	No
	A plantar incision in the proximal phalanx	3.56	5.00	1.81	No
6	Beaver 64 scalpel blade	4.22	5.00	1.39	No
	Beaver 64 MIS scalpel blade	4.00	5.00	1.73	No
7	The same scalpel	4.33	5.00	0.87	No
	A blunt elevator	3.11	4.00	2.03	No
8	The same scalpel	2.78	2.00	1.56	No
	A blunt elevator	3.33	4.00	1.87	No
9	A Shannon-Isham burr of an appropriate size	5.00	5.00	0.00	No
10	Manual pressure	4.56	5.00	0.88	No
	A rasp	4.78	5.00	0.67	No
11	Suture thread	2.56	3.00	1.59	Yes
	Closure strips	1.89	1.00	1.36	Yes
	Bandage pressure	3.00	2.00	1.94	Yes

**Table 4:** Delphi round 3 results

Items	Statement	Mean	Median	SD
3	A longitudinal incision to the distal pulp of the toe plus a second longitudinal incision to the centre of the plantar aspect of the head of the proximal phalanx	4.44	5.00	1.13
11	Suture thread	4.44	5.00	1.13
	Closure strips	1.89	1.00	1.36
	Bandage pressure	3.00	2.00	1.94

**Fig. 1:**
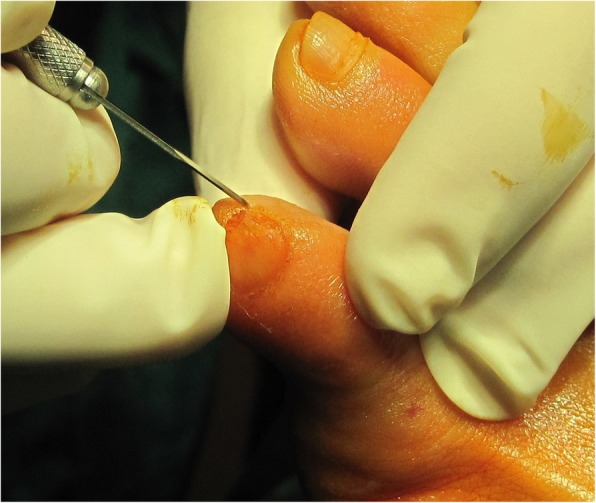
Incision to the distal pulp of the toe

**Fig. 2:**
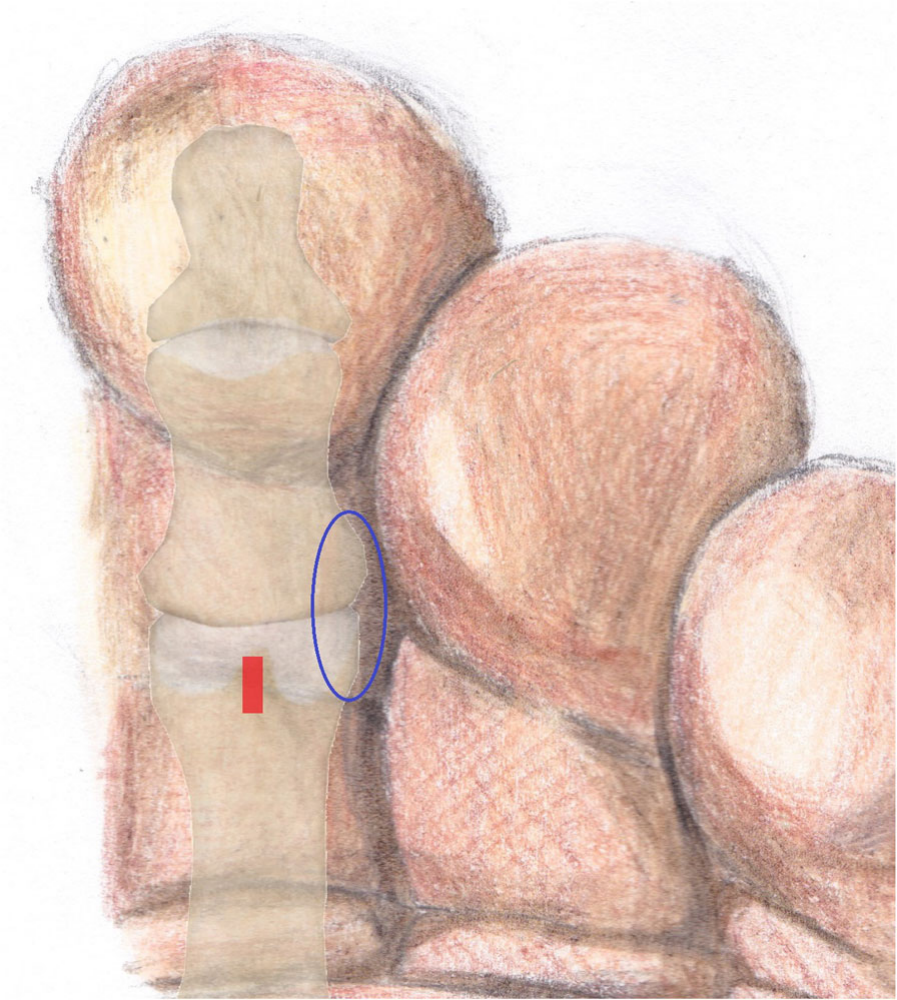
Incision to the centre of the plantar aspect of the head of the proximal phalanx

**Fig. 3:**
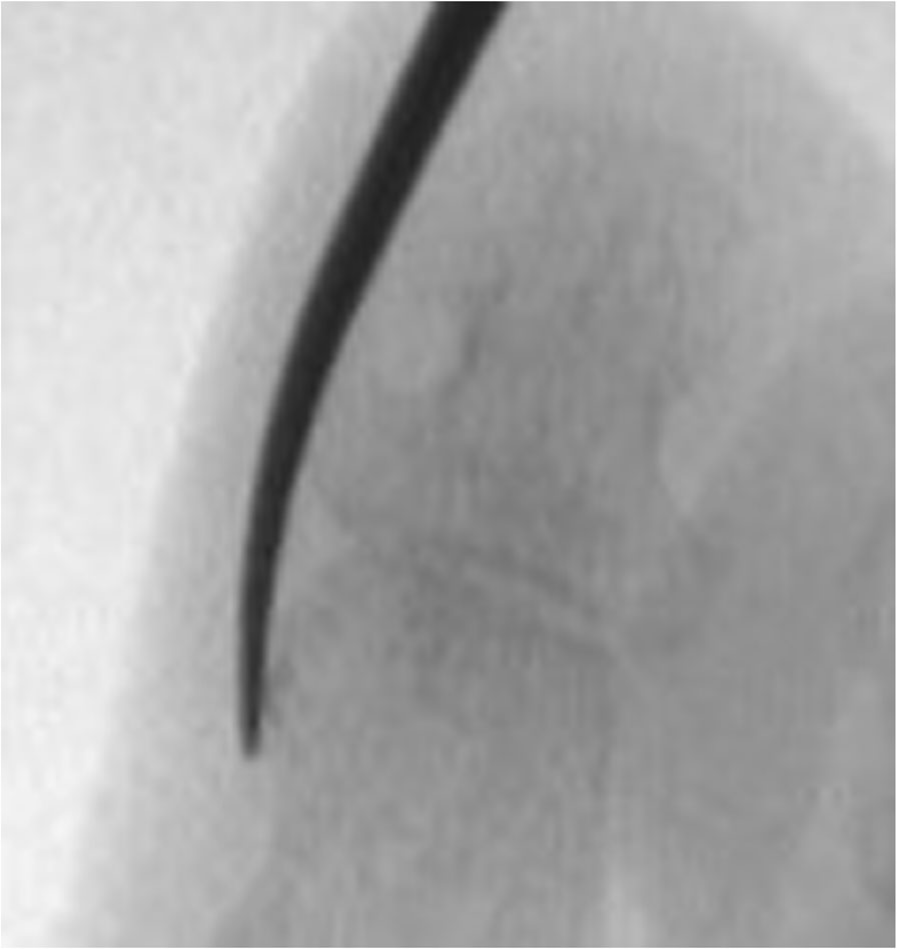
A blunt elevator for separating adhesions and periosteal elevation

**Fig. 4:**
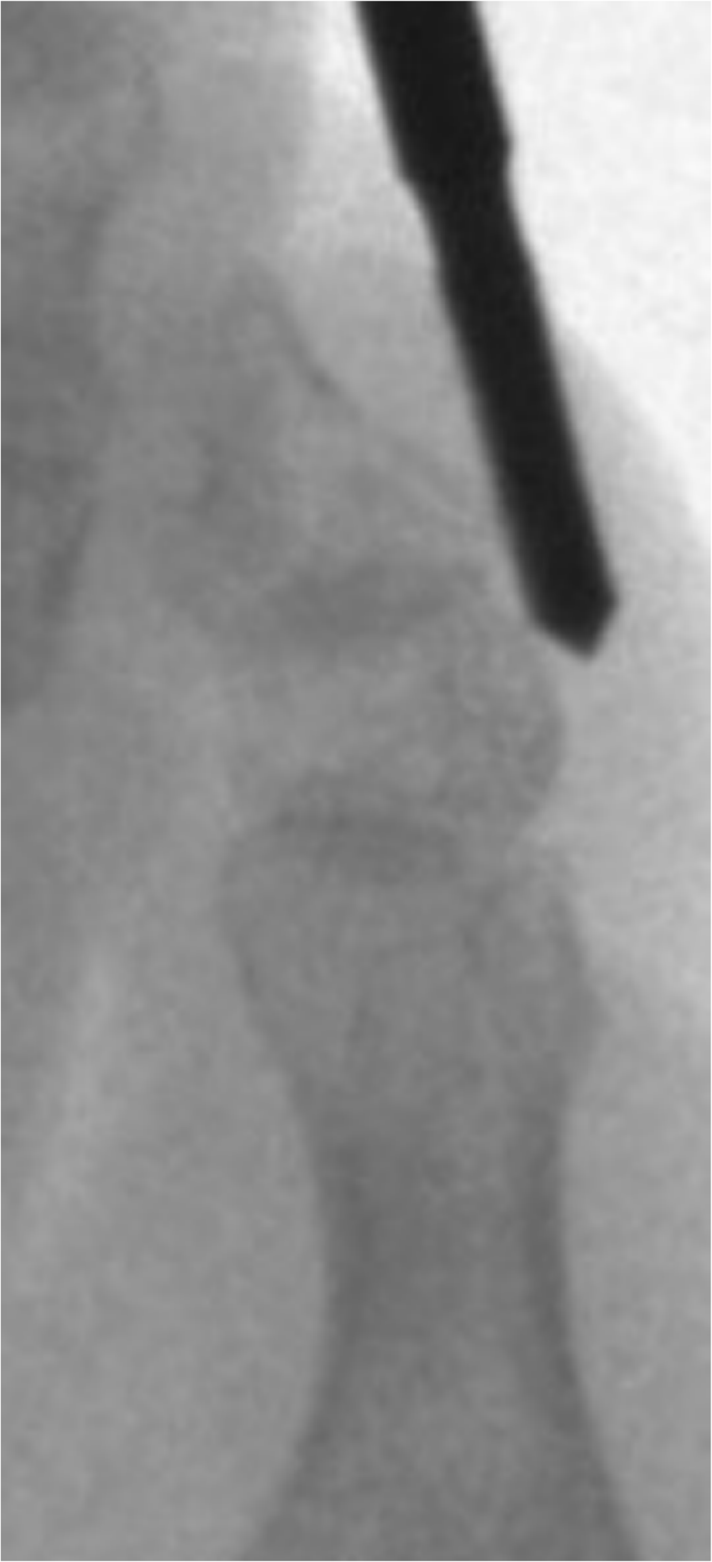
Location of the Shannon-Isham burr

**Fig. 5:**
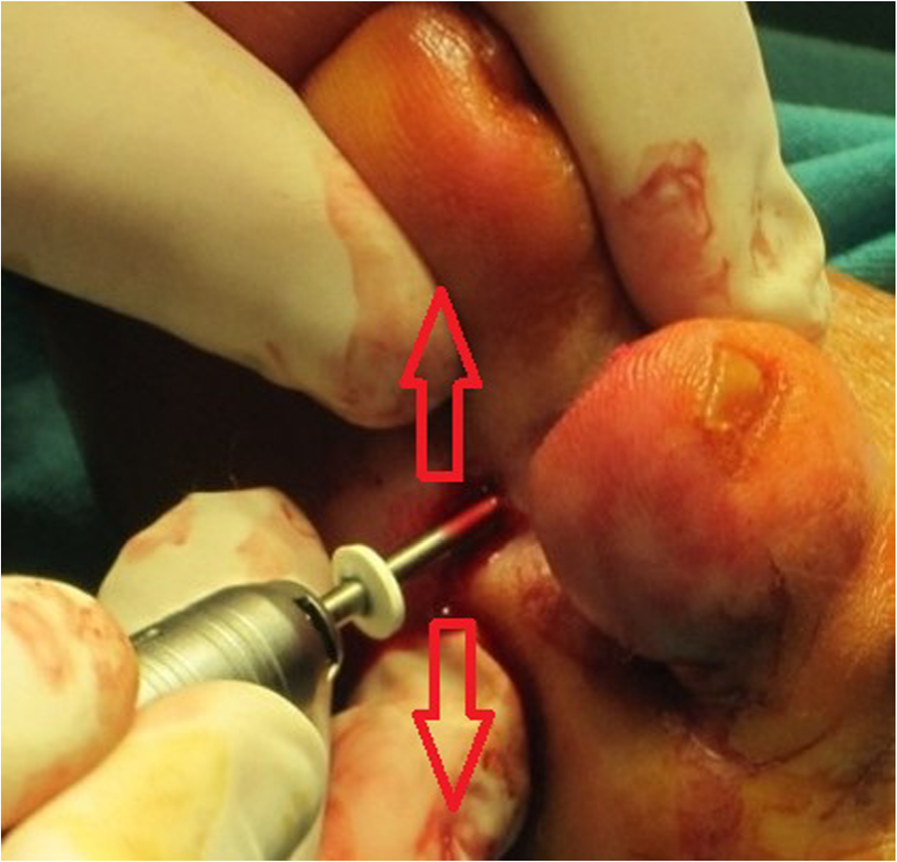
An osteotripsy with Shannon-Isham burr

After the analysis of the final results, a final report was issued that included the statements that received the highest agreement. The following recommendations for MIS on the interdigital helomas of the lesser toes were created based on this consensus:

- A longitudinal incision to the distal pulp of the toe is most acceptable for the resection of the exostosis or the hypertrophic medial or lateral condyle of the distal phalanx (Fig. 1).
- A longitudinal incision to the distal pulp of the toe is most acceptable for the resection of the exostosis or the hypertrophic medial or lateral condyle that simultaneously affects the distal phalanx and the middle phalanx on the same side.
- A longitudinal incision to the distal pulp of the toe plus a second longitudinal incision to the centre of the plantar aspect of the head of the proximal phalanx is the most acceptable technique for the resection of the exostosis or the hypertrophic medial or lateral condyle that simultaneously affects the distal phalanx and the proximal phalanx on the same side.
- An incision to the centre of the plantar aspect of the head of the proximal phalanx is most acceptable for the resection of the exostosis or the hypertrophic medial or lateral condyle that affects the head of the proximal phalanx of the lesser toes (Fig. 2).
- An incision to the centre of the plantar aspect of the head of the proximal phalanx is most acceptable for the resection of the exostosis or the hypertrophic medial or lateral condyle that simultaneously affects the head of the proximal phalanx and the middle phalanx on the same side of the lesser toes.
- A Beaver 64 scalpel blade is the most acceptable instrument for performing a minimally invasive skin incision on the interdigital heloma of the lesser toes.
- The same scalpel is the most acceptable tool for extending the incision until contact with the bone.
- A blunt elevator is the most acceptable instrument for separating adhesions and periosteal elevation (Fig. 3).
- A Shannon-Isham burr of an appropriate size is the most acceptable for the bone area with regard to performing an osteotripsy (Figs. 4 and 5).
- Extracting the bone paste with a rasp is the most acceptable option.
- The closure of the incision with suture thread is the most acceptable option.

## Discussion

This study provides recommendations based on an expert consensus regarding the performance of a condylectomy via MIS on the interdigital heloma of the lesser toes. Foot surgeons and researchers could use these recommendations to guide their clinical practice or research.

Given that all of the experts used a local anaesthesia technique for the affected toe only and performed the surgery without ischaemia, we assumed that these decisions were the most appropriate to perform this technique and, therefore, these issues were not included in the questionnaire. Likewise, a fluoroscope was a commonly used intraoperative control element of the surgical technique because of its low emission of radiation compared with conventional X-rays.

For the condylectomy of the lateral or medial condyle of the distal phalanx, either separate from or associated with the medial or lateral condylectomy of the middle phalanx, a longitudinal incision in the distal pulp of the toe is recommended, in agreement with the methods of Gorman et al. [17], De Prado et al. [19], and Bycura [18]. This approach preserves the plantar neurovascular bundles and maintains a sufficient distance from the bony prominence to be resected to enable an adequate range of motion to perform the condylectomy. By contrast, White [15] proposed a longitudinal plantar incision in the sagittal plane over the bony prominence and lateral to the flexor tendon. This approach might compromise the plantar digital neurovascular bundle because, as we indicated, one part is located on the sides of the plantar aspect. Hymes [16] proposed a longitudinal dorsal incision to the sagittal plane over the bony prominence, lateral to the extensor tendon and proximal to the bony area to be resected. However, this approach can compromise the dorsal digital neurovascular bundle.

Regarding the condylectomy incision of the lateral or medial aspect of the head of the proximal phalanx, either separate from or associated with the lateral or medial condylectomy of the middle phalanx, it should be performed longitudinally along the sagittal plane in the centre of the plantar aspect of the head of the proximal phalanx. This approach crosses the flexor tendon but does not produce tenotomy of the flexor tendon, nor does it affect its functionality. In addition, the neurovascular bundles are preserved, which on the plantar aspect are located on the sides. This approach does not coincide with the proposals of any of the reference authors. White [15], Gorman et al. [17], Bycura [18], and De Prado et al. [19] proposed a longitudinal plantar incision along the sagittal plane over the bony prominence and lateral to the flexor tendon; however, this pathway can compromise the plantar digital neurovascular bundle, so that the risk of causing sequelae is greater than if the incision approaches the flexor tendon. Hymes [16] proposed a longitudinal dorsal incision along the sagittal plane, lateral to the extensor tendon, and proximal to the head of the proximal phalanx; however, this pathway can also compromise the dorsal digital neurovascular bundle.

In agreement with White [15], the panel of experts recommended using the Beaver 64 scalpel, which is 2 mm wide and has a curved distal end with a cut that facilitates dissection, so that it will be uniform in size throughout its entire course until it comes into contact with the bone. However, the scalpels proposed by the other authors have drawbacks. Hymes [16] recommended a number 15 that is 3 mm wide; as such, the incision will be larger, and the tissues will receive more trauma than is necessary. Gorman et al. [17] and Bycura [18] proposed using the Beaver 67 scalpel. This scalpel is the same size as the Beaver 64 scalpel, but its distal tip is pointed; as such, the dissection will not have a uniform size throughout its entire length. Alternatively, De Prado et al. [19] recommended the use of the Beaver 64 MIS scalpel, which is 1 mm wide and smaller than that of the burrs and rasps, which might cause the tissue to tear, leaving a bruise on the skin and a worse prognosis for healing.

None of the reference authors described how to separate adhesions and perform periosteal elevation. The current consensus-based study recommends that it be conducted with a blunt elevator to not risk damaging nearby neurovascular structures.

To perform the condylectomy, White [15] indicated the use of a Shannon burr but did not detail the model or size. Hymes [16] and Bycura [18] recommended a Shannon 44 burr for all cases; however, this size could be excessive when treating interdigital helomas. De Prado et al. [19] proposed a short Shannon 44 burr, which has a more suitable size for most cases but will not be sufficient for certain cases. However, the results of the current study indicate that the best option is to use the Shannon-Isham burr of an appropriate size for the bone surface being resected, as recommended by Gorman et al. [17].

The reference authors [16, 20] proposed manual pressure and/or washing with physiological saline solution to extract the bone paste. In addition, Hymes [16] and De Prado et al. [19] added the use of a rasp. However, our panel of experts recommended only the use of a rasp, placing the edges of the rasp in contact with the bone and then in contact with the soft tissue, so that the bone paste remains deposited on the edges of the rasp. Washing with physiological saline solution in an incision of such small size might compromise the total extraction of the bone paste.

The panel of experts recommended closing the incision with a suture thread, as proposed by all of the reference authors with the exception of Hymes [16], who proposes using closure strips. However, their ability to maintain an approximation of the wound edge is less than when using suture thread.

### Limitations

First, the Delphi method is not exempt from the possible influence of the researchers because they participated in the development of the questionnaire, the analysis of the partial results, and the editing of the final report. Second, the criteria used to select the experts might not have adequately identified participants with sufficient clinical experience. However, we believe that we selected the professionals with the most experience in this field. In addition, the participation of different types of specialists (podiatrists and traumatologists) enriched this study, offering different points of view. Third, the results of this study might not reflect the opinions of experts from around the world because eight participants were from Spain, and only two additional countries were represented by the remaining two experts. Although the number of panellists is small, a small sample can be considered effective when the experts have similar levels of training and a general understanding of the field of interest [30]. Fourth, the line of progression given when evaluating the proposed statements might have led the experts to give the highest scores when expressing their agreement with the statement, despite it not being what they would perform in clinical practice, because they nevertheless consider it valid. Therefore, this aspect might introduce information bias. Finally, expert opinion is known as level V of evidence-based medicine but this type of study which collects the experience of experts is needed to improve the quality of treatment for patients [32].

## Conclusions

Through the Delphi consensus, we obtained a detailed and precise description of the surgical technique for condylectomy with MIS to treat interdigital helomas on the lesser toes. These findings allow us to have a well-defined and regulated procedure to execute this surgical technique in a standardised manner and therefore obtain reproducible results. In addition, this information might be useful in the preparation of clinical trials evaluating the effectiveness of this technique to treat interdigital helomas on the lesser toes.

## Abbreviations

AAFAS: Academy of Ambulatory Foot & Ankle Surgery
AEMIS: Asociación Española de Cirugía de Mínima Incisión del Pie
MIS: Minimally invasive surgery
